# Nutritional Supplementation Concurrent with Nutrition Education Accelerates the Wound Healing Process in Patients with Diabetic Foot Ulcers

**DOI:** 10.3390/biomedicines8080263

**Published:** 2020-08-03

**Authors:** Raedeh Basiri, Maria T. Spicer, Cathy W. Levenson, Michael J. Ormsbee, Thomas Ledermann, Bahram H. Arjmandi

**Affiliations:** 1 Department of Nutrition Food and Exercise Sciences, Florida State University, Tallahassee, FL 32306, USA; mspicer@my.fsu.edu (M.T.S.); mormsbee@fsu.edu (M.J.O.); 2 Center for Advancing Exercise and Nutrition Research on Aging, Florida State University, Tallahassee, FL 32306, USA; 3 Dietetics and Nutrition Program, Keiser University, Lakeland, FL 32306, USA; 4 Department of Biomedical Sciences, College of Medicine, Florida State University, Tallahassee, FL 32306, USA; cathy.levenson@med.fsu.edu; 5 Institute of Sports Sciences & Medicine, Florida State University, Tallahassee, FL 32306, USA; 6 Discipline of Biokinetics, Exercise and Leisure Sciences, School of Health Sciences, University of KwaZulu-Natal, Durban 4000, South Africa; 7 Department of Family and Child Sciences, Florida State University, Tallahassee, FL 32306, USA; tledermann@fsu.edu

**Keywords:** diabetes, diabetic foot ulcers, nutrition supplementation, nutrition education, wound healing, malnutrition, antioxidants

## Abstract

Trials on nutritional supplements for the treatment of diabetic foot ulcer (DFU) have only evaluated the effects of supplementation with specific nutrients. Additionally, nutrition education has not been a systematic part of these studies. The aim of this study was to evaluate the effects of a nutrient-dense formula combined with nutrition education on wound healing in DFU patients. Twenty-nine patients were randomly assigned to the treatment group (*n* = 15) receiving two servings of supplements daily plus nutrition education or control group (*n* = 14) that received the standard of care but no additional nutritional or educational intervention. Both groups were followed for a maximum of 12 weeks. Wound healing, as measured by planimetry, was examined at baseline and every four weeks until complete wound closure or up to 12 weeks. There were no significant differences between groups for BMI, age, duration of diabetes, wound age estimation, or wound area at baseline. The treatment group experienced a faster wound healing rate (6.43 mm^2^/week more reduction in the wound area) than the control group. The mean reduction in the wound area during the first four weeks of the study was almost 13-fold greater in the treatment group compared to the control group (18.0 mm^2^/week vs. 1.4 mm^2^/week, respectively). Our findings showed that nutrition supplementation plus nutrition education significantly accelerated wound healing in DFU patients compared to those who just received a standard-of-care regimen.

## 1. Introduction

Diabetic foot ulcers (DFU) are chronic wounds in the foot or feet associated with neuropathy and/or peripheral arterial disease of the lower limb in patients with diabetes mellitus (DM). Ten to fifteen percent of patients with DM may develop DFU, which in some cases can lead to lower-extremity amputation [1]. Reports from the Centers for Disease Control and Prevention (CDC) show that in the United States, there are nearly one in four adults living with diabetes, which indicates that a large number of Americans are at risk of DFU [2]. The total medical cost of managing DFU ranges from $9 to $13 billion in addition to the cost of DM management alone [3]. Improvements in wound care therapy would not only help with the financial burden of DFU but also increase life expectancy and quality of life in these patients.

Evidence has shown that patients with DFU are at high risk of malnutrition. A study by Maier et al. evaluated dietary intake of DFU patients and showed that they only met 55% of the dietary reference intakes (DRI) for energy (*p* = 0.008) and consumed 0.57 g protein/kg body weight (BW), which is alarmingly lower than the DRI for adults (0.8 g protein/kg BW) [4]. They also had a significantly (*p* < 0.05) lower intake of micronutrients such as vitamin E, B1, B2, B3, B6, magnesium, calcium, iron, potassium, and sodium, which are needed for wound healing. Similarly, Sajid et al. [5] showed that protein intake in males and females with DFU was significantly lower than the recommendations (76.9 g and 56.8 g compared to 219.5 g and 130.2 g, respectively). Malnutrition or inadequate intake of essential nutrients involved in wound healing worsens wound severity and increases wound complications such as infection and amputation which may lead to death. There is a high prevalence of malnutrition in patients with DFU. In fact, it has been reported that 62% of patients with DFU are malnourished and this is closely correlated with the severity of infection (r = 0.64, *p* < 0.001) [6]. The incidence of developing a moderate or severe infection was approximately 70% in malnourished DFU patients, while it was only 5% in well-nourished patients. Gau et al. [7] reported that 14.6% of DFU patients were malnourished and 70.5% were at risk for malnutrition. Malnutrition was correlated with increased lower-extremity amputation (*p* = 0.001) and the severity of amputation (*p* < 0.01). The rate of major amputations (above the ankle) was almost 11-fold higher in the malnourished group compared to those who were well-nourished (15.5% vs. 1.4%, respectively). Although the high prevalence of malnutrition and its negative effects on the healing of DFU is reported in several studies [4,5,6,7], to our knowledge there is no study looking at the effects of complete nutrition supplementation. Studies in this field have only examined the effects of supplementing diabetic patients with arginine, glutamine, beta-hydroxy-beta-methylbutyrate (HMB), magnesium, and vitamin E [8,9,10]. Furthermore, nutrition education has rarely been incorporated in DFU studies with supplementation. Educating DFU patients about their increased dietary needs as well as the best dietary sources of essential nutrients involved in wound healing can increase patients’ knowledge and awareness and improve their dietary intake. Adequate dietary intake of essential nutrients can improve wound healing by ameliorating poor outcomes associated with malnutrition. The aim of this study was to investigate the effects of nutrition supplementation concurrently with nutrition education on the rate of wound healing in patients with DFU.

## 2. Experimental Section

### 2.1. Screening and Recruitment

This study was approved by the Florida State University Institutional Review Board on 14 June 2016 and renewed on 11 May 2017, 25 April 2018, and 2 April 2019 (HSC# 2016.18608, HSC#2017.21018, HSC#2018.23474, HSC# 2019.26726) and Tallahassee Memorial Hospital (TMH) Institutional Review Board on 28 September 2016 and renewed on 25 September 2017, 6 July 2018 and 7 June 2019 (IRB # 2016-10). The trial was registered at clinicaltrials.gov NCT04055064. To maintain the confidentiality of personal health information, a point-of-contact person from the clinic was identified, educated about the study’s primary inclusion/exclusion criteria as described later. The contact person explained the study to potential participants and referred them to the researcher for screening if they were inclined to participate in the study. Inclusion criteria were male or non-pregnant, non-lactating female of ages between 30 and 70 years old, diagnosed with type 1 or type 2 diabetes; undergoing pharmacological treatment for glycemic control, with at least one foot ulcer of grade 1A based on University of Texas (UT) wound classification [11]. Exclusion criteria were hemoglobin A1c (HbA1c) concentration >12%, use of bioengineered tissue within the last four weeks, a history of radiation treatment to the ulcer site, immunosuppressed (e.g., having HIV or shingles), active malignancy, chronic kidney disease, liver failure/cirrhosis, heart failure and/or myocardial infarction in the past three months, use of warfarin, excessive alcohol use according to World Health Organization standards, or any mental or physiological condition that may interfere with nutrition education and nutritional supplement intake.

### 2.2. Study Design and Data Collection

In a randomized control study with repeated measures, patients who met the inclusion criteria were informed of the details of the study and consented if they were willing to participate. All patients, irrespective of their grouping, received standard wound care from the TMH Wound Care Clinic. One group of patients, chosen randomly, received nutritional supplements concurrently with nutrition education in addition to the standard-of-care regimen, while another group received only the standard-of-care regimen. At baseline, dietary intake, wound size, and anthropometric measurements were assessed by one of the trained research staff. Participants were visited in the clinic every two weeks for wound evaluation/monitoring, receiving supplements, and nutrition education. Anthropometric measurements, dietary intake, and wound area were assessed every four weeks and/or at the time of complete wound closure. As the wound approached complete healing, participants were monitored weekly to ensure measurement at the correct endpoint.

### 2.3. Intervention/Treatment

Patients in the treatment group were instructed to consume two servings (474 mL) of a nutrient-dense formula, Boost Glucose Control, which was purchased from Nestle (Nestle HealthCare Nutrition, Inc., Bridgewater, NJ, USA), between meals—preferably one in the morning between breakfast and lunch and one in the afternoon between lunch and dinner for 12 weeks or until the complete occurrence of wound closure. The supplement was designed for diabetic patients and contained at least 50% of the RDA recommendations for essential nutrients. The treatment group was also educated about improving their diet by consuming more low-fat high-quality protein sources, vegetables, complex carbohydrates, and less simple carbohydrates. This was carried out by the researcher through explaining different food groups and giving examples of the best food choices in each food group. The education took approximately 10 min for the first session and approximately five minutes every four weeks to repeat and emphasize the educational material, answer possible questions, and encourage patients to continue choosing better food options. Two servings of supplements provided a total of 500 kilocalories, 28 g of protein, and essential vitamins and minerals. A complete list of the nutrient content of the supplement is shown in Appendix A, Table A1. To increase adherence to the intervention, patients chose from three different flavors (vanilla, strawberry, and chocolate). Table 1 shows the recommended daily allowance (RDA) for nutrients involved in wound healing and compares it with the nutrient content of two servings of the supplements. Our goal was to provide patients with adequate supplements so that they can receive at least 50% of the RDA recommendations for essential nutrients. We hypothesized that nutrition education would improve the dietary intake of nutrients and motivate participants to meet the remaining 50% of nutrient recommendations by consuming better food sources.

### 2.4. Measurements

Body weights were measured at baseline and every four weeks thereafter using a stand-on scale (Seca Mechanical Column Scale, Hamburg, Germany). Height was self-reported and body mass index (BMI) was calculated using weight in kilograms divided by height in meters squared. To evaluate HbA1c, we used HbA1c Now+ by PTS Diagnostics which has been validated for use in clinical settings [12].

### 2.5. Dietary Assessment

Dietary intake data were recorded using 24 h recall. Participants were questioned about all the food and beverages consumed during the last 24 h every four weeks up to 12 weeks or until the complete wound closure. Nutrient intake was estimated using the newest version of Food Processor SQL version 11.1.480 (ESHA’s Food Processor^®^, Salem, OR, USA). Nutrients included in the analysis were total energy, protein, vitamin A, vitamin C, vitamin E, zinc, copper, and manganese since studies have shown that they were most effective in wound healing [13,14]. Daily energy and protein requirements were set based on the National Pressure Ulcer Advisory Panel (NPUAP) guidelines [15], 30–35 kcal/kg of body weight and 1.2–1.5 g/kg of body weight, respectively. Energy and protein intakes of patients were compared with those of NPUAP recommendations and the micronutrient intake of patients was compared with DRIs. We used (dietary intake of each nutrient by participants/recommendation for that nutrient) × 100 to show the percentage of the recommended nutrient met by the participants.

### 2.6. Wound Measurement and Characterization

Wound dimensions were assessed using the PictZar^®^ wound planimetry software program (Advanced Planimetric Services, L.L.C., Elmwood Park, NJ, USA). Wound pictures were taken using a Sony digital camera (Cyber-shot DSC-RX100; Sony Corporation, Tokyo, Japan) and then uploaded in the software. The wound was photographed with a ruler placed near the wound which was used for calibration of the linear dimensions of the image. Wound sizes including length, width, and area were estimated by the PictZar^®^ software after the wound dimensions and border was manually detected and traced with a computer mouse. The wound healing rate was calculated using the following formula to standardize change in the area.

### 2.7. The Wound Healing Rate = (Current Area-Baseline Area)/Time (Number of Weeks) Endpoints

The primary endpoint was the change in the area of the wound at 12 weeks and the secondary endpoint was complete healing.

### 2.8. Statistical Analysis

Data were analyzed using the Statistical Package for Social Science (SPSS) version 25.0 (SPSS, Inc., Chicago, IL, USA). The statistical significance value was set at *p* < 0.05 for all tests. Descriptive statistics were used to evaluate population characteristics and independent-samples t-test was used to compare means of confounding variables between groups at baseline. One-way ANOVA with repeated measures was conducted to assess changes in the area of the wound and to determine differences among groups with posthoc Bonferroni’s correction for multiple comparisons. To eliminate the effects of gender, HbA1C, wound age estimation, and duration of diabetes these factors used as cofactors in the statistical analyses.

### 2.9. Data and Resource Availability

The datasets generated from this study are available from the corresponding author upon reasonable request.

## 3. Results

**Figure 1:**
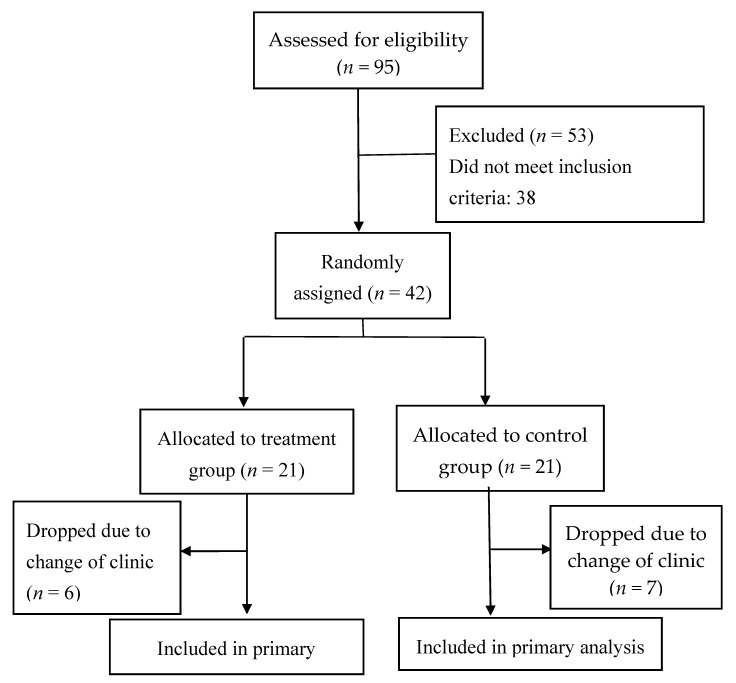
Study flow diagram.

Ninety-five (95) patients were screened but only 42 patients met the inclusion criteria. Patients were then randomly assigned to either the treatment or control group. Figure 1 shows the study flow diagram.

### 3.1. General Characteristics

Descriptive data of relevant characteristics are outlined in Table 2.

Baseline characteristics in both groups were similar. The age of the study population was 53.3 ± 11.1 years (mean ± SD). All patients had a wound of either grade 1 or 2 stage A based on the UT wound classification. The mean wound area in both groups was similar at baseline [Treatment: 45 ± 11 (mm^2^); Control: 45 ± 15 (mm^2^)]. There was no statistically significant difference in age, ethnicity, body mass index (BMI), hemoglobin A1C, duration of diabetes, or wound age estimation between participants in the treatment and control groups at baseline. To assess normality, the Kolmogorov– Smirnov test was used and the results indicated that all the continuous variables did not significantly deviate from the normal distribution, with the exception of the wound age estimation (*p* < 0.05). Distribution of gender was different in the treatment and control group but there were no significant gender differences within each group for the wound healing rate (*p* = 0.2). The mean duration of diabetes in the treatment group was 14.4 ± 8 years and in the control group 11.7 ± 6 years; however, the difference was not statistically significant. There were no significant differences between the groups regarding indicators of socioeconomic status (SES) and other factors involved in nutritional status including cultural and religious dietary restrictions, appetite problems, and previous unintentional weight loss. Indicators of SES were living alone, having financial support, being employed, having financial concerns, and food needs. Having routine visits with a registered dietitian (RD) was not part of the standard care for this population and only seven patients in the treatment and three patients in the control group had visited RDs once in the past. There was no increase in the concentrations of hemoglobin A1C (HbA1C) in the treatment or the control group during the study period. In the treatment group, mean HbA1C was 7.95% (63 mmol/mol) at baseline and 7.67% (60 mmol/mol) at the end of the study. The control group had a mean HbA1c of 8.40% (68 mmol/mol) at baseline and 8.01% (65 mmol/mol) at the end of the study.

### 3.2. Dietary Intake of Participants at Baseline

**Figure 2:**
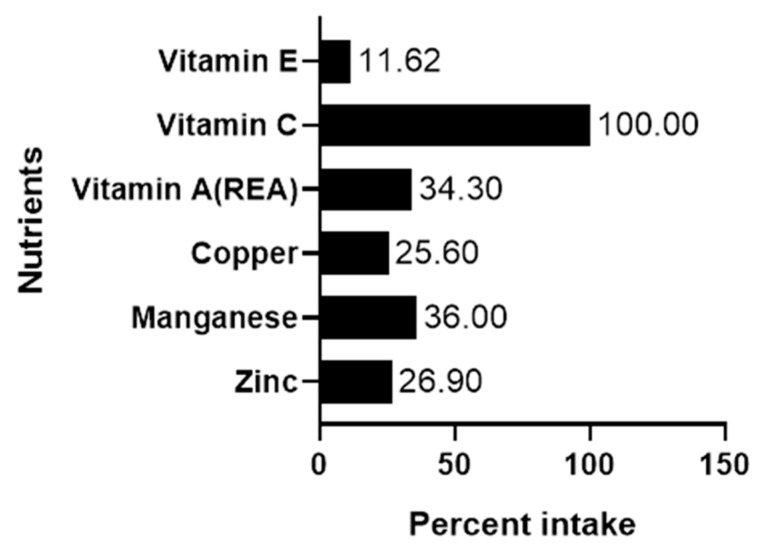
Mean percent nutrient intake in DFU patients. Numbers show the percentage intake compared to Dietary Recommended Intake at baseline.

At baseline, the mean percentage of energy and protein intakes of participants compared to the National Pressure Ulcer Advisory Panel (NPUAP) recommendation were alarmingly low (50% and 48.7%, respectively). Figure 2 demonstrates percentage of micronutrient intake compared to DRI recommendation for all participants at baseline. The mean dietary intake of zinc, manganese, copper, vitamin A, and vitamin E were alarmingly lower than the DRI with the exception of vitamin C which met the requirement for a healthy population.

### 3.3. Change in Dietary Intake of Participants During the Study

Comparison of energy intake of patients with NPUAP recommendations showed that both treatment and control groups had a low dietary intake of energy at baseline (mean% ± SD = 53.2% ± 22.6 and 42.7% ± 16.8, respectively). The difference between the energy intake of the treatment and the control group at baseline was not statistically significant. The effects of confounding factors such as gender, age, living alone, being employed, and wound age estimation on energy intake was examined. Only being employed had a significant effect on energy intake (*p* = 0.03) and therefore was set as a covariate in the model. The interaction between group and time was not statistically significant for energy intake after adjustment for confounding factors; however, the mean energy intake of the treatment group reached 70.5% of the recommendations during the study.

**Figure 3:**
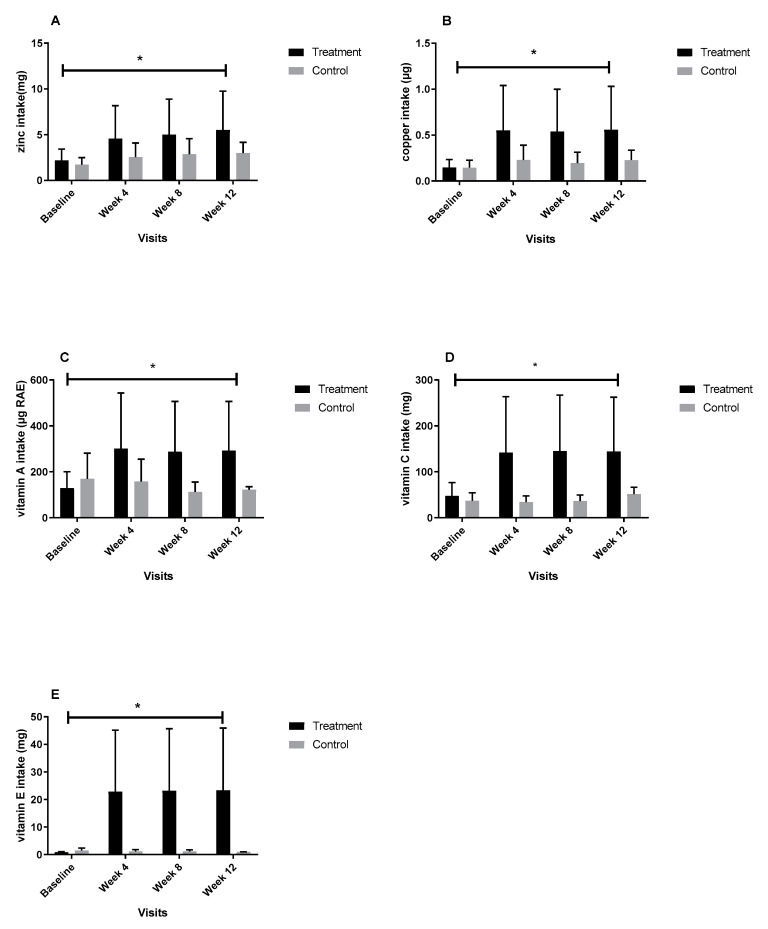
(**A**–**E**). Intake of zinc (**A**), copper (**B**), vitamin A (**C**), C (**D**), and E (**E**) during the 12 weeks of follow up. The numbers for the control group show intake from diet and for treatment group show a combination of intakes from two servings of supplements and diet. The difference between the mean zinc intake of the two groups was statistically significant in weeks four and eight and twelfth of the study (*p* = 0.02). The interaction between group and time was statistically significant for copper, vitamin A, C, and E (*p* < 0.001, *p* = 0.001, *p* < 0.001, *p* < 0.001, respectively). Bars represent the means ± SEM. * Denotes a significant time by group interaction (*p* ≤ 0.05).

In comparison with NPUAP, both treatment and control groups had a low intake of protein (mean% ± SD = 54.5% ± 24.7 and 43.1% ± 18.2, respectively). The mean intake of protein at baseline was not statistically significant between the two groups. The effects of confounding factors (similar to the energy intake) on the intake of protein were examined; however, none of the factors had significant effects on the protein intake of the patients. Therefore, the model was run without any covariate. The interaction between time and group was not statistically significant for protein intake; however, the mean protein intake of the treatment group could reach 84.9% of the NPUAP recommendations. Dietary intake of essential micronutrients was significantly improved in the treatment group during the follow up. Figure 3A–E shows the micronutrient intake of the treatment and control groups at different time points of the study. Dietary intake of zinc, copper, vitamins A, C, and E increased significantly in the treatment group while there were no significant changes in the nutrient intake of the control group during the follow up. Dietary intake of vitamin A was decreased in the control group during the 12 weeks of the follow up. The interaction between group and time was not statistically significant for dietary intake of manganese.

### 3.4. Comparison of the Wound Healing Rate between Groups

Nine patients from the treatment group and 10 patients from the control group experienced complete wound closure during the study. Although more patients from the control group experienced complete wound closure, the mean reduction in wound area in the treatment group was 6.43 mm^2^/week more than the control group. The mean reduction in DFU area during the first four weeks of the study was 18.01 mm^2^/week in the treatment group compared to 1.4 mm^2^/week in the control group (*p* = 0.01).

**Figure 4:**
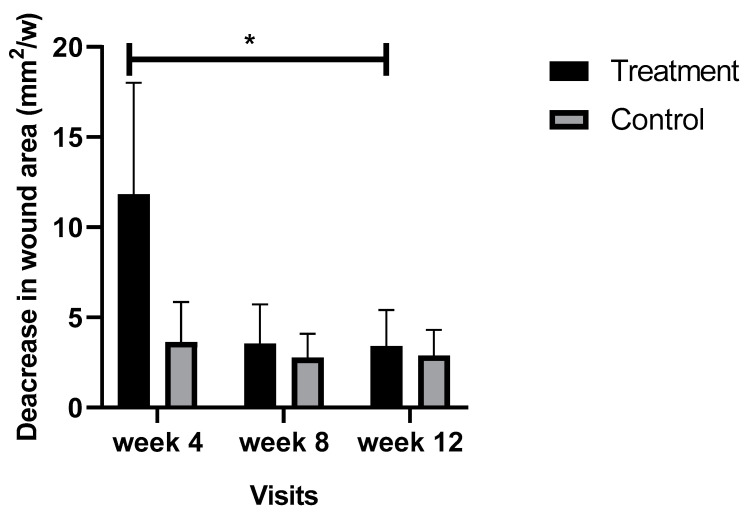
Comparison of the healing rate during the 12 weeks of the study period. Bars represent the means ± SEM. * Denotes a significant time by group interaction (*p* ≤ 0.05).

Comparison of the healing rate at different time points of the study for the treatment and control groups are outlined in Figure 4. The mean wound areas in the treatment and control group were similar at baseline (45 ± 11 mm^2^ and 45 ± 15 mm^2^, respectively). The greatest effects of the intervention were during the first four weeks of the study, which the mean healing rate in the treatment group was 12.85-fold faster than the control group (*p* = 0.01).

## 4. Discussion

Our findings demonstrate that nutrition intervention along with education is beneficial for the treatment of DFU in undernourished patients when added to optimized local wound care. These results support the findings of similar studies which showed a decrease in cost of antibiotic use and an improvement in wound appearance and depth score [8,9]. The greatest effect of the intervention on the wound healing rate was during the first four weeks of the current study. The decreased wound healing rate during the second and third four weeks of the study could be due to loss of power as a result of losing participants due to the complete wound closure. The strong positive effects of supplementation can be synergistic as a result of the appropriate intake of all the nutrients together since studies on supplementing with only one or two nutrients showed less positive effects [10,16]. While similar treatments have been effective in other types of chronic wound healing such as pressure ulcers [17,18], to our knowledge, there has not been any study conducted on the effects of complete supplementation along with nutrition education on wound healing in DFU patients. Therefore, our findings are novel and should be considered as a model of treatment.

We analyzed the dietary intake of energy, protein, vitamins C, E, and A as well as manganese, copper, and zinc in both groups of the study. Adequate energy and protein intake are required for maintaining energy balance and supporting a positive nitrogen balance. Protein is also required for optimum wound healing and is essential for the synthesis of enzymes, which are involved in healing, cell proliferation, and collagen synthesis. Vitamin C plays an important role in immunomodulation, collagen synthesis, and antioxidant status and, therefore, is an essential nutrient in different phases of wound healing such as inflammation, proliferation, and remodeling [14]. Vitamin E is well known for its antioxidant activity, which helps with limiting the generation of reactive oxygen species (ROS) [19]. Controlling the reactions initiated by ROS can be very helpful in the prevention of prolonged inflammation. Biological properties of vitamin A related to wound healing are antioxidant activity and modulation of cellular proliferation and differentiation. Vitamin A increases fibroblast proliferation, collagen cross-linkage, synthesis of hyaluronate, and decreases matrix metalloproteinase mediated extracellular matrix degradation [20]. Manganese and copper are part of the superoxide dismutase (SOD), one of the most important antioxidant complexes in our body, which can help with regulating the high levels of ROS in DFU patients [20]. Zinc is involved in antioxidant function (part of SOD), cell replication, tissue repair, nucleic acid metabolism and growth, and protein synthesis [21]. The results of this study showed that dietary intake of energy, protein, and essential micronutrients for wound healing were severely low in patients with DFU even when they were compared with the recommendations for a healthy population [22]. These findings are consistent with other studies that reported significant low dietary intake of energy, protein, and micronutrients in this population [4,7]. Our results also demonstrated that the intervention applied in the present study enhanced the dietary intake of micronutrients in the treatment group which could be a reason for the impressive wound healing rate observed in this population. There was no significant change in the micronutrient intake of patients in the control group.

Although participants in this study had a high BMI, which classified them as overweight or obese, their intakes of energy, protein, and essential micronutrients involved in wound healing were alarmingly lower than the minimum recommendations by NPUAP (30 kcal/kg, 1.2 g/kg) and DRI, respectively. The low dietary intake can be due to lack of knowledge, low income, physical inability to purchase or prepare healthy food, depression, and/or recommendations for restricted diets by medical staff due to a combination of chronic diseases that this population is dealing with [3,23,24]. In the current study, we did not control for dietary intake hence it can be suggested that following the regimen that was used in this study, patients with DFU can benefit from liberalized diets. Once the wound is completely healed energy restrictions may be gradually implemented as needed [25]. Due to a scarcity of recommendations for micronutrient intake in DFU patients, we compared the micronutrient intake of participants with recommendations for a healthy population although this population has higher needs for antioxidants due to the oxidative stress and presence of an open wound [13,19,20,21,26,27,28,29,30,31].

We standardized the wound healing rate by measuring the decrease in the area of the wound per week to reduce the bias in our analysis. Similar studies [17,32] measured only percentage change in wound area or time to complete wound closure, which can be biased due to the different sizes of the wounds in different patients. The strength of our study was that energy, protein and essential micronutrients were given to the participants as complete nutrition and within the context of appropriate nutritional care. We educated patients about a healthy diet to increase their knowledge and awareness while giving them supplements to help them meet their requirements easier. The supplements were easy to consume and participants had a choice to choose their favorite flavor which resulted in the high adherence to the treatment. Supplements were tolerated well and no adverse events reported.

This study had several limitations. Due to the small population in the area, we were not able to assess the effects of supplementation or nutrition education independently. The education intervention in this study was general and we were not able to offer individualized dietary recommendations. There were no recommendations for dietary intake of DFU patients to be used for the assessment of the adequacy of energy, protein, and micronutrient intake in this population. We were not able to design a specific supplement for this population; however, we chose the best available commercially manufactured supplement to meet most of the dietary needs of our participants. All the participants were recruited from one clinic, which could affect the results of the study due to patients communicating with each other.

Future research should identify the effectiveness of individualized diets in combination with social and psychological interventions. Routine visits with a dietitian are essential for nutritional assessment and appropriate individualized recommendations and can help patients to improve and/or maintain overall nutritional status which results in positive clinical outcomes. Identifying adequate dietary intake of energy, protein and micronutrients in DFU, especially obese and overweight, patients is essential and can make a big difference in the quality of life and cost of treatment in this population. Nutrition intervention is a cost-effective [33,34] approach in the treatment of DFU and should be considered as an integral part of wound care in DFU patients. In conclusion, nutrition supplementation combined with education can accelerate wound healing in patients with DFU.

## Figures and Tables

**Table 1:** Comparison of nutrient intake from two servings of supplement with RDA for age and gender.

Nutrient	Total from Supplements/Day	RDA for Nutrient	% of RDA Provided; Men vs. Women if RDA Varied for Men vs. Women
Protein	28 g	56 g 46 g	50% 61%
Vitamin C	204 mg	60 mg	304%
Vitamin E	66 IU	33.3 IU	200%
Vitamin A	2500 IU	3000 IU	83%
Zinc	6 mg	men: 11 mg women: 8 mg	54% 75%
Copper	0.8 mg	0.9 mg	88%
Manganese **	0.8 mg	men: 2.3 mg women: 1.8 mg	35% 44%

IU: international unit; ** Numbers are showing adequate intake (AI), as there is no established RDA for manganese.

**Table 2:** Baseline characteristics of participants by group.

Groups	Treatment	Control	*p*-Value
N	15	14	--
Men/women	8/7	11/3	--
Age (year)			
Means ± SD	52.93 ± 9.74	53.79 ± 12.84	
Median	54	55	0.84
Interquartile range	12	21	
Ethnicity			
African American	4	3	0.75
White	11	11	
BMI * (kg/m^2^)			
Means ± SD	33.54 ± 7.98	34.07 ± 6.04	
Median	31.1	34.5	0.84
Interquartile range	16.7	18.4	
Diabetes duration (years)			
Means ± SD	14.40 ± 8.03	11.71 ± 6.17	0.32
Median	12	12	
Interquartile range	10	12	
Wound age estimation (months)			
Means ± SD	10.97 ± 15.09	10.58 ± 18.27	
Median	6	6	0.95
Interquartile range	10	9.5	
HbA1C **			
Means ± SD	7.95 ± 2.06	8.40 ± 2.16	
Median	7.3	8.8	0.57
Interquartile range	2.5	4.4	
Dietary restrictions (yes/no)	2/13	1/13	1
Appetite problem (yes/no)	1/14	1/13	1
Visited RD *** (yes/no)	7/8	3/11	0.3
Unintentional weight loss (yes/no)	4/11	1/13	0.32
living alone (yes/no)	3/12	4/10	0.6
Having financial support (yes/no)	11/4	11/3	0.75
Employed (yes/no)	4/11	6/8	0.37
Having financial concern (yes/no)	6/9	3/11	0.3
Need food (yes/no)	2/13	1/13	0.6
Smoke (yes/no)	3/12	3/12	1

BMI = body mass index; HbA1C = hemoglobin A1C; RD = registered dietitian.

## Appendix A

**Table A1:** Nutrient composition of 237 mL of the nutritional supplement.

Nutrient	Amount
Calories (kcal)	250
Calories from fat (kcal)	110
Total Fat (g)	12
Saturated Fat (g)	1.5
Trans Fat (g)	0
Cholesterol (mg)	<5
Sodium (mg)	270
Potassium (mg)	260
Total Carbohydrate (g)	23
Dietary Fiber (g)	3
Sugars (g)	6
Protein * (g)	14
Vitamin A ** (IU)	1250
Vitamin C (mg)	102
Calcium (mg)	250
Iron (mg)	3.6
Vitamin D (IU)	240
Vitamin E (IU)	33
Vitamin K (mcg)	16
Thiamin (mg)	0.3
Riboflavin (mg)	0.34
Niacin (mg)	4
Vitamin B6 (mg)	0.4
Folic Acid (mcg)	80
Vitamin B12 (mcg)	1.2
Biotin (mcg)	60
Pantothenic Acid (mg)	2
Phosphorus (mg)	200
Iodine (mcg)	30
Magnesium (mg)	80
Zinc (mg)	3
Selenium (mcg)	14
Copper (mg)	0.4
Manganese (mg)	0.4
Chromium (mcg)	24
Molybdenum (mcg)	15
Chloride (mg)	204
L-carnitine (mg)	25
Taurine (mg)	20
Inositol (mg)	200

* Includes protein from caseinate and L-arginine; ** Includes 45% of vitamin A activity from betacarotene.
